# 异基因造血干细胞移植后出血患者预后分析和预测模型构建

**DOI:** 10.3760/cma.j.issn.0253-2727.2022.06.007

**Published:** 2022-06

**Authors:** 嘉乾 戚, 涛 尤, 虹 王, 伟 韩, 祎 范, 佳 陈, 德沛 吴, 悦 韩

**Affiliations:** 苏州大学附属第一医院、江苏省血液研究所、国家血液系统疾病临床医学研究中心、苏州大学造血干细胞移植研究所、血液学协同创新中心，苏州 215006 The First Affiliated Hospital of Suzhou University, Jiangsu Institute of Hematology, National Center for Hematology and Clinical Research, Hematopoietic Stem Cell Transplantation Institute of Soochow University, Hematology Collaborative Innovation Center, Suzhou 215006, China

**Keywords:** 异基因造血干细胞移植, 出血, 预测模型, Allogeneic hematopoietic stem cell transplantation, Hemorrhage, Forecasting model

## Abstract

**目的:**

研究发生异基因造血干细胞移植（allo-HSCT）相关出血患者的预后并构建出血预测模型。

**方法:**

回顾性分析苏州大学附属第一医院2004年5月1日至2012年4月1日期间接受allo-HSCT的555例恶性血液病患者的临床资料并构建出血事件预测模型。

**结果:**

在全部555例患者中302例（54.0％）发生出血事件，其中Ⅰ度出血151例（27.0％），Ⅱ度出血63例（11.0％），Ⅲ度出血48例（9.0％），Ⅳ度出血40例（7.0％）。多因素分析显示，出血等级较高（Ⅲ、Ⅳ度）患者的总死亡率（*HR*＝12.53，95％*CI* 7.91～19.87，*P*<0.001）及非复发死亡率（*HR*＝23.79，95％*CI* 12.23～46.26，*P*<0.001）均较高于低出血等级组。另外，供者合并心脑血管疾病、移植物抗宿主病（GVHD）分度、血小板重建不良以及血小板无效输注与出血风险呈现独立相关。通过以上变量构建的出血模型展现了较好的准确度（C-index＝0.934），其效能比既往出血模型具有明显优势。

**结论:**

allo-HSCT患者发生高等级（Ⅲ/Ⅳ度）出血事件后死亡风险增加。经交叉验证的出血风险预测模型对其出血事件的预测和提前干预具有一定价值。

## Abstract (en)

**Objective:**

To study hematopoietic stem cell transplantation-related bleeding prognosis and construct a bleeding prediction model.

**Methods:**

The clinical data of 555 patients with malignant hematologic diseases who underwent allogeneic hematopoietic stem cell transplantation between May 1^st^ 2004, and April 1^st^ 2012 was analyzed retrospectively, and a prediction model was constructed.

**Results:**

Of the 555 patients, a total of 302 (54.0％) patients exhibited bleeding events of varying degrees, including 151 (27.0％) with grade Ⅰ bleeding, 63 (11.0％) with grade Ⅱ bleeding, 48 (9.0％) with grade Ⅲ bleeding, and 40 (7.0％) with grade Ⅳ bleeding. Multifactorial analysis showed that the overall mortality (*HR*＝12.53, 95％*CI* 7.91–19.87, *P*<0.001) and non-recurrence mortality (*HR*＝23.79, 95％*CI* 12.23–46.26, *P*<0.001) were higher in patients with higher bleeding grades (Ⅲ and Ⅳ bleeding) compared to those with lower bleeding grades. Additionally, the donor's underlying disease, graft-versus-host disease (GVHD) score, poor platelet reconstitution, and ineffective platelet transfusion were independently associated with bleeding risk. The bleeding model constructed using the above variables showed good accuracy (C-Index＝0.934), and its efficacy was significantly higher than previous bleeding models.

**Conclusion:**

Hematopoietic stem cell transplant patients are at increased risk of death after a bleeding event. The cross-validated bleeding risk prediction model is valuable for early intervention.

造血干细胞移植是血液恶性肿瘤的有效治疗方法，出血性并发症是移植后患者的重要预后不良因素[1]。造血干细胞移植后发生胃肠道出血、弥漫性肺泡出血（DAH）和颅内出血患者的死亡率明显增高[1]–[3]。本研究中，我们通过回顾性分析，确定移植后出血对异基因造血干细胞移植（allo-HSCT）患者总死亡率（OM）、非复发死亡率（NRM）和累积复发率（CIR）的影响，并据此构建移植后出血预测模型。

## 病例与方法

1. 病例：2004年5月1日至2012年4月1日期间在苏州大学附属第一医院接受allo-HSCT的555例患者纳入回顾性队列。排除正在接受抗血栓治疗、合并严重肾脏/肝脏功能障碍及先天性出血性疾病、缺乏出血性并发症信息、没有随访信息的患者。记录患者的年龄、性别、诊断、原发疾病、恢复状况、髓外浸润、基因突变、器官功能损伤、移植类型、干细胞来源、GVHD预防措施、GVHD分度、粒细胞重建、血小板重建、血小板无效输注、输注细胞数量、抗胸腺细胞球蛋白（ATG）应用、尿常规、BK病毒感染、巨细胞病毒（CMV）感染、其他病原体感染以及出血事件情况。所有患者或其直系亲属均知情同意。研究方案通过苏州大学伦理委员会审核批准［（2012）伦研批第196号］。

2. 出血等级的定义：依据世界卫生组织（WHO）量表[4]确定出血等级：① 0度：没有出血。② Ⅰ度：瘀斑/紫癜，或稀疏/不融合的口咽部出血，鼻出血<30 min。③ Ⅱ度：呕血、咯血、新鲜便血、肌肉骨骼出血或软组织出血，发病后24 h内不需要输红细胞且无血流动力学不稳定。④ Ⅲ度：呕血、咯血、血尿（包括无血块的间歇性大出血）、异常阴道出血、新鲜便血、鼻出血和口咽部出血、侵入部位出血、肌肉骨骼出血或软组织出血，在发病后24 h内需要输红细胞且无血流动力学障碍。⑤Ⅳ度：造成身体虚弱的出血（包括视网膜出血和视力损伤）以及其他导致血流动力学不稳定的出血。0～Ⅱ度出血被定义为低等级出血，Ⅲ、Ⅳ度被定义为高等级出血。

3. 随访：随访采用电话随访的方式，随访截止日期为2021年1月1日，中位随访时间为62（17～67）个月。

4. 统计学处理：统计分析使用R studio（版本3.6.0）和rms、PredictABLE、ggplot2、survminer软件包。正态分布和偏态分布的连续变量分别以“均值±标准差”和“中位数（四分位距）［*M*（IQR）］”表示，并使用对应使用非配对*t*检验和Mann-Whitney *U*检验进行比较。分类变量以百分比表示，并使用卡方检验进行比较。累积率用Kaplan-Meier曲线显示，并使用Log-rank检验进行比较。单因素和多因素生存分析使用Cox比例危险模型进行评估。使用Fine-Gray模型来评估竞争性终点的风险，包括NRM和累积复发率（CIR）。使用森林图来显示不同变量对预后的影响。使用Harrell's c-Index、校准曲线和重分类测试来确定出血因素超越其他因素所赋予预后的能力，并计算净重新分类指数（NRI）和整体鉴别指数（IDI）。绘制与时间相关的接受者特征曲线及面积（AUROC），以评估不同模型的预后效果。通过多因素Logistics回归筛选变量并应用提名图将构建的模型可视化。使用校准曲线法评估模型的一致性。使用决策曲线分析评估了该模型与传统出血评分相比的净临床效益。还利用机器学习方法，包括随机森林和决策树，通过训练集的交叉验证来评估模型的预测准确性。

## 结果

1. 患者疾病临床特征：纳入本研究的555例患者中，男332例（59.8％），女223例（40.2％），中位移植年龄34（23, 42）岁，急性髓系白血病（AML）218例（39.3％），急性淋巴细胞白血病（ALL）137例（24.7％），慢性髓性白血病（CML）126例（22.7％），非霍奇金淋巴瘤（NHL）28例（5.0％），骨髓增生异常综合征（MDS）36例（6.5％），急性混合细胞白血病8例（14.4％），毛细胞白血病2例（0.4％），其他临床特征详见表1。共有302例（54.0％）患者发生出血事件，其中Ⅰ度出血151例（27.0％），Ⅱ度出血63例（11.0％），Ⅲ度出血48例（9.0％），Ⅳ度出血40例（7.0％）。

**表1:** 555例接受异基因造血干细胞移植患者的一般资料和临床特征

指标	结果
移植前疾病状态［例（％）］	
CR	372（67.0）
NR	183（33.0）
髓外浸润［例（％）］	61（11.0）
遗传学改变［例（％）］	190（34.2）
器官功能损伤［例（％）］	142（25.6）
患者合并心脑血管疾病［例（％）］	137（24.7）
供者合并心脑血管疾病［例（％）］	82（14.8）
供者类型［例（％）］	
同胞全相合	321（57.8）
单倍型	64（11.5）
无关供者	147（26.5）
脐血干细胞	23（4.1）
造血干细胞来源［例（％）］	
骨髓	230（41.4）
外周血干细胞	240（43.2）
骨髓+外周血干细胞	85（15.3）
输注CD34^+^细胞数［×10^6^/L，*M*（IQR）］	4.21（3.12, 5.58）
输注单个核细胞数［×10^8^/L，*M*（IQR）］	7.82（5.51, 10.61）
移植预处理方案［例（％）］	
BU/CY	408（73.5）
其他	147（26.5）
血小板无效输注［例（％）］	114（20.5）
急性GVHD［例（％）］	
未发生+Ⅰ度	297（53.5）
Ⅱ度	122（22.0）
Ⅲ度	48（8.6）
Ⅳ度	88（15.9）
粒细胞重建良好［例（％）］	541（97.5）
血小板重建良好［例（％）］	450（81.1）
细菌或真菌感染［例（％）］	319（57.5）
尿常规异常［例（％）］	149（26.8）
CMV感染［例（％）］	70（12.6）
EB病毒感染［例（％）］	130（23.4）
BK病毒感染［例（％）］	104（18.7）

注：IQR：四分位距；BU：白消安；CY：环磷酰胺；CR：完全缓解；NR：未缓解；CMV：巨细胞病毒

2. 生存分析：单因素生存分析显示，高等级出血是OM的危险因素（*HR*＝19.14，95％*CI* 12.12～30.22，*P*<0.001）。Kaplan-Meier曲线显示，与低等级出血患者相比，高等级出血患者的5年OS率降低（*P*<0.001）（图1A）。Kaplan-Meier生存曲线显示，与Ⅱ度级以下出血的患者相比，Ⅲ度以上出血患者的NRM（图1B）明显增加（*P*<0.001）。此外，基因突变、器官功能损伤、供者或患者合并心脑血管疾病、发生血小板无效输注、发生重度GVHD、血小板重建不良、尿常规异常、细菌或真菌感染与总生存率相关（表2）。多变量调整后，Ⅲ度以上出血可显著增加患者死亡风险（*HR*＝12.53，95％*CI* 7.91～19.87，*P*<0.001）（表3）。由于复发和死亡终点在生存分析中是相互竞争的，因此对NRM和CIR拟合了Fine-Gray竞争风险模型。结果显示，Ⅲ度以上出血对非复发死亡有预测作用（*HR*＝111.99，95％*CI* 38.59～325.02，*P*<0.001）。经多变量调整后，Ⅲ度以上出血仍然是非复发死亡的独立危险因素（*HR*＝23.79，95％*CI* 12.23～46.26，*P*<0.001）。值得注意的是，在多变量分析中，Ⅲ度以上出血是NRM和OS的最强风险因素（表3）。

**图1:**
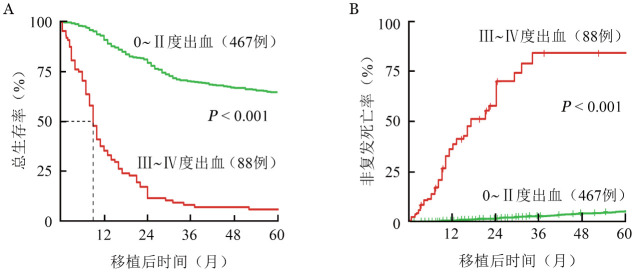
造血干细胞移植移植后不同出血等级患者的总生存曲线（A）和非复发死亡曲线（B）

**表2:** 555例接受异基因造血干细胞移植患者移植后5年OS、DFS、NRM及CIR影响因素的单因素分析

变量	OS		DFS		NRM		CIR	
	*HR*（95% *CI*）	*P*值	*HR*（95% *CI*）	*P*值	*HR*（95% *CI*）	*P*值	*HR*（95% *CI*）	*P*值
年龄（≥45岁，<45岁）	1.21（0.88~1.68）	0.240	0.90（0.56~1.45）	0.669	1.50（0.96~2.34）	0.072	0.73（0.56~0.96）	0.024
性别（男，女）	0.92（0.72~1.19）	0.544	0.93（0.67~1.31）	0.681	0.95（0.66~1.38）	0.791	0.98（0.81~1.19）	0.832
诊断								
ALL，AML	0.76（0.56~1.04）	0.087	0.83（0.56~1.24）	0.374	0.73（0.46~1.15）	0.176	0.98（0.79~1.21）	0.839
CML，AML	0.55（0.39~0.78）	0.001	0.75（0.49~1.13）	0.165	0.38（0.21~0.67）	0.001	1.10（0.88~1.36）	0.404
其他，AML	0.67（0.52~0.86）	0.002	0.87（0.62~1.22）	0.431	0.53（0.37~0.76）	0.001	1.04（0.85~1.26）	0.728
移植前状态（CR，NR+PR）	0.84（0.65~1.09）	0.199	1.11（0.77~1.60）	0.562	0.63（0.44~0.92）	0.016	0.89（0.72~1.09）	0.258
髓外浸润（是，否）	1.72（1.21~2.46）	0.003	1.52（0.92~2.49）	0.099	1.82（1.10~3.01）	0.020	1.17（0.85~1.60）	0.341
遗传学异常（是，否）	2.56（1.99~3.29）	<0.001	3.39（2.42~4.75）	<0.001	1.68（1.16~2.44）	0.006	2.14（1.73~2.63）	<0.001
器官功能损伤（有，无）	0.41（0.29~0.58）	<0.001	0.69（0.46~1.02）	0.064	0.11（0.04~0.26）	<0.001	1.05（0.85~1.28）	0.661
患者基础疾病（有，无）	3.49（2.71~4.50）	<0.001	5.33（3.80~7.47）	<0.001	1.75（1.17~2.61）	0.006	1.74（1.39~2.18）	<0.001
供者基础疾病（有，无）	2.78（2.07~3.74）	<0.001	1.97（1.28~3.04）	0.002	3.49（2.33~5.22）	<0.001	1.49（1.10~2.02）	0.009
移植供者类型								
单倍型供者，同胞相合供者	0.99（0.66~1.47）	0.952	0.55（0.28~1.08）	0.082	1.47（0.89~2.43）	0.132	0.98（0.72~1.33）	0.889
无关供者，同胞相合供者	1.29（0.99~1.70）	0.062	1.38（0.97~1.97）	0.077	1.28（0.86~1.90）	0.227	1.04（0.84~1.29）	0.696
脐血，同胞相合供者	2.36（1.46~3.81）	<0.001	1.08（0.44~2.65）	0.860	3.70（2.08~6.61）	<0.001	0.81（0.43~1.52）	0.511
造血干细胞来源								
外周血，骨髓	2.03（1.58~2.61）	<0.001	1.35（0.97~1.88）	0.077	3.43（2.32~5.09）	<0.001	0.88（0.73~1.07）	0.217
外周血+骨髓，骨髓	1.18（0.84~1.65）	0.339	1.42（0.93~2.16）	0.102	0.81（0.46~1.41）	0.454	1.30（1.00~1.67）	0.047
CD34^+^细胞（≥中位数，<中位数）	1.00（0.78~1.28）	0.985	0.93（0.67~1.30）	0.673	1.09（0.75~1.56）	0.654	1.05（0.87~1.27）	0.611
单个核细胞（≥中位数，<中位数）	1.04（0.81~1.34）	0.749	1.06（0.76~1.48）	0.718	1.00（0.70~1.44）	0.995	1.11（0.92~1.34）	0.267
移植前预处理（BU/CY，其他）	0.97（0.74~1.29）	0.856	1.20（0.81~1.77）	0.375	0.78（0.53~1.16）	0.227	1.05（0.84~1.30）	0.670
血小板无效输注（是，否）	1.64（1.24~2.17）	<0.001	1.40（0.96~2.06）	0.082	1.76（1.18~2.63）	0.005	1.06（0.83~1.36）	0.616
急性GVHD分度								
Ⅱ度，未发生+Ⅰ度	1.68（1.22~2.31）	0.001	1.19（0.80~1.76）	0.398	3.31（1.90~5.78）	<0.001	1.02（0.80~1.29）	0.887
Ⅲ度，未发生+Ⅰ度	2.47（1.63~3.73）	<0.001	1.21（0.66~2.21）	0.545	6.58（3.54~12.22）	<0.001	0.68（0.46~0.99）	0.046
Ⅳ度，未发生+Ⅰ度	3.25（2.35~4.49）	<0.001	0.82（0.45~1.51）	0.533	10.53（6.38~17.37）	<0.001	1.06（0.76~1.47）	0.746
粒细胞重建（是，否）	0.65（0.34~1.27）	0.211	0.68（0.28~1.66）	0.394	0.69（0.26~1.88）	0.469	0.87（0.47~1.64）	0.676
血小板重建（是，否）	0.35（0.26~0.46）	<0.001	0.98（0.61~1.60）	0.947	0.17（0.12~0.24）	<0.001	1.01（0.75~1.36）	0.954
出血分级								
Ⅰ级，0级	2.29（1.61~3.26）	<0.001	2.10（1.45~3.02）	<0.001	6.80（2.21~20.87）	0.001	0.96（0.78~1.18）	0.687
Ⅱ级，0级	3.64（2.42~5.47）	<0.001	1.13（0.62~2.08）	0.688	39.47（13.80~112.85）	<0.001	0.52（0.36~0.75）	<0.001
Ⅲ级，0级	9.79（6.49~14.75）	<0.001	1.97（0.99~3.91）	0.054	111.99（38.59~325.02）	<0.001	1.31（0.74~2.32）	0.354
Ⅳ级，0级	19.14（12.12~30.22）	<0.001	0.95（0.23~3.96）	0.940	187.21（63.38~553.02）	<0.001	0.59（0.22~1.59）	0.298
尿常规异常（是，否）	2.49（1.93~3.21）	<0.001	1.20（0.82~1.77）	0.343	4.75（3.28~6.87）	<0.001	0.94（0.74~1.19）	0.602
CMV感染（是，否）	0.81（0.55~1.20）	0.299	0.59（0.32~1.06）	0.077	1.12（0.67~1.87）	0.671	0.99（0.74~1.32）	0.940
EB病毒感染（是，否）	1.00（0.71~1.41）	0.984	1.06（0.68~1.65）	0.807	0.86（0.51~1.45）	0.566	1.13（0.88~1.45）	0.340
BK病毒感染（是，否）	2.62（1.99~3.44）	<0.001	2.09（1.43~3.05）	<0.001	3.10（2.11~4.55）	<0.001	1.09（0.84~1.43）	0.515
细菌与真菌感染（是，否）	1.50（1.15~1.94）	0.003	1.05（0.75~1.46）	0.795	2.28（1.51~3.46）	<0.001	0.92（0.76~1.11）	0.361

注：OS：总生存；DFS：无病生存；NRM：非复发死亡率；CIR：累积复发率；ALL：急性淋巴细胞白血病；AML：急性髓系白血病；CML：慢性髓性白血病；GVHD：移植物抗宿主病；BU：白消安；CY：环磷酰胺；CR：完全缓解；NR：未缓解；CMV：巨细胞病毒。遗传学改变指在不同疾病中存在对预后有不良影响的相关染色体异常、基因突变及融合基因

**表3:** 造血干细胞移植后5年总生存（OS）、非复发死亡率（NRM）的多变量生存分析

变量类型	OS		NRM	
	*HR*（95%*CI*）	*P*值	*HR*（95%*CI*）	*P*值
年龄（≥45岁，45岁）	0.93（0.67~1.31）	0.692	1.41（0.87~2.28）	0.164
性别（男，女）	0.79（0.61~1.02）	0.071	0.83（0.56~1.22）	0.334
髓外浸润（是，否）	1.17（0.78~1.75）	0.446	0.93（0.52~1.67）	0.809
器官功能损伤（有，无）	0.70（0.48~1.02）	0.062	0.27（0.11~0.67）	0.005
患者合并心脑血管疾病（有，无）	6.24（4.56~8.54）	<0.001	4.20（2.50~7.07）	<0.001
供者合并心脑血管疾病（有，无）	1.63（1.15~2.32）	0.007	1.64（0.95~2.82）	0.076
血小板无效输注（是，否）	1.63（1.22~2.18）	0.001	2.22（1.44~3.42）	<0.001
血小板重建（是，否）	1.14（0.76~1.69）	0.528	1.08（0.64~1.85）	0.765
粒细胞重建（是，否）	1.24（0.60~2.57）	0.568	1.38（0.47~4.04）	0.561
尿常规异常（是，否）	1.26（0.91~1.74）	0.166	1.68（1.04~2.72）	0.035
CMV感染（是，否）	0.59（0.38~0.91）	0.016	0.80（0.44~1.44）	0.450
EB病毒感染（是，否）	1.47（1.01~2.12）	0.042	2.21（1.22~4.02）	0.009
BK病毒感染（是，否）	1.29（0.93~1.78）	0.125	0.86（0.53~1.41）	0.558
细菌与真菌感染（是，否）	0.91（0.68~1.22）	0.538	1.26（0.77~2.05）	0.355
移植类型（同胞全相合移植，其他）	0.83（0.62~1.11）	0.208	0.85（0.55~1.30）	0.447
细胞来源（外周血干细胞，其他）	0.85（0.62~1.17）	0.326	1.19（0.73~1.92）	0.492
出血等级（Ⅲ~Ⅳ度，0~Ⅱ度）	12.53（7.91~19.87）	<0.001	23.79（12.23~46.26）	<0.001
GVHD分度（Ⅱ~Ⅳ度，未发生+Ⅰ度）	1.28（0.95~1.73）	0.101	2.27（1.35~3.82）	0.002

注：CMV：巨细胞病毒

3. 预测模型的构建、验证与效能评估：为了证实出血等级对不同终点的独立预测价值，我们进行了模型分析。结果显示，在基本模型中加入出血等级后，Cox回归模型预测OM的AUC显著增加［0.79（0.75～0.83）对0.86（0.82～0.90），*P*<0.01］（图2A），Fine-gray模型预测NRM的AUC也显著增加［0.88（0.84～0.92）对0.92（0.88～0.96），*P*<0.01］（图2B）。在常规因素的基础上增加出血等级可改善预测OM（NRI＝0.058，*z*＝2.448，*P*＝0.014；IDI＝0.08，*z*＝2.201，*P*＝0.028）和NRM（NRI＝0.182，*z*＝3.962，*P*<0.001；IDI＝0.202，*z*＝3.785，*P*<0.001）。重新分类表显示，在基本多变量预测模型中加入出血等级后，区分所有风险类别的OM（表4）和NRM（表5）患者的情况有所改善。

**图2:**
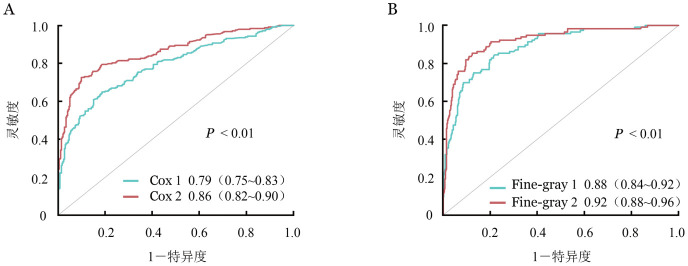
Cox回归模型预测总死亡率（A）和Fine-gray模型预测非复发死亡的AUC曲线（B）

**表4:** 总死亡率（OM）的基本预测模型中加入出血等级后的重新分类表

重新分类		更新后模型			重分类比例（％）
初始模型	风险等级	低	中	高	
	低	330	16	10	10
	中	50	38	24	68
	高	10	14	63	22

**表5:** 非复发死亡（NRM）的基本预测模型中加入出血等级后的重新分类表

重新分类		更新后模型			重分类比例（％）
初始模型	风险等级	低	中	高	
	低	267	25	3	12
	中	70	71	11	64
	高	2	36	70	24

出血作为造血干细胞移植后的一个致命事件，其对多种终点均显示出重要的预后价值。对出血的预测能够改善造血干细胞移植患者的预后。鉴于此，我们建立了一个与出血相关的多变量风险评分模型。首先，从整个研究人群中随机抽样，分别得出372例患者的训练集和183例患者的测试集。训练集的单变量分析得出疾病状态、器官功能损伤、患者或供者合并心脑血管疾病、外周血干细胞输注、ATG、GVHD预防、GVHD阶段、粒细胞重建、血小板重建、EBV感染和细菌或真菌感染是出血的风险因素。进而我们通过LASSO回归筛选了GVHD阶段、供者基础疾病、血小板无效输注和尿检异常纳入模型。为了量化每个协变量对出血等级的贡献，我们生成了提名图来可视化。从该模型中得出的新的出血评分显示出良好的校准度（C-Index＝0.934）（图3）。与已建立的出血评分相比，包括改良门诊患者出血风险指数（mOBRI）、HAS-BLED和肝肾及肿瘤疾病出血评分量表（HEMORR2），我们构建的新模型具有更大的临床效益。测试集的交叉验证显示了该模型的高准确性和精确性（图4A）。此外，机器学习模型，包括分类树（图4B）和随机森林（图4C）显示了与Logistic模型相似的出血风险预测功效。最后，使用生存分析进一步验证了出血评分的预后能力。根据167分的尤登指数分级，与出血分数较低的患者相比，Ⅲ度以上出血患者具有较高的OM（*HR*＝29.75，95％*CI* 10.24～86.39，*P*<0.001）和NRM（*HR*＝167.21，95％*CI* 24.77～1128.74，*P*<0.001）。

**图3:**
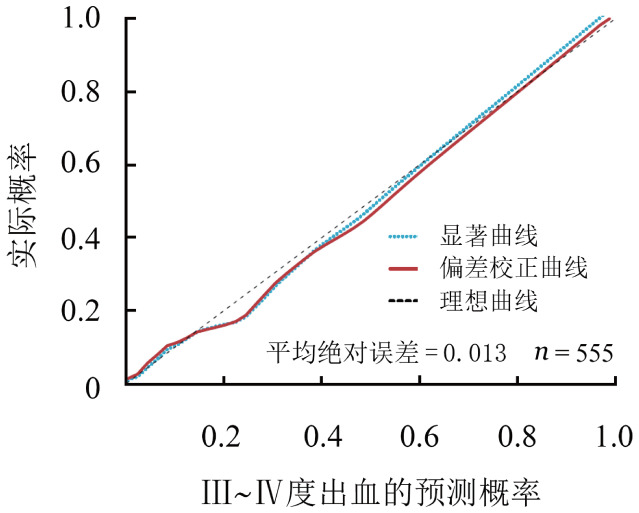
造血干细胞移植患者高等级出血模型的预测能效

**图4:**
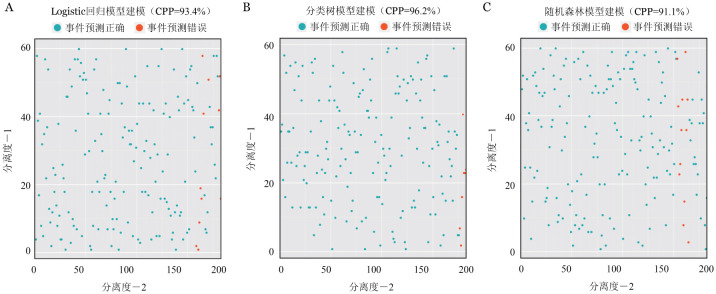
Logistic回归模型（A）、分类树模型（B）、随机森林模型（C）的预测准确性可视化 CPP：预测准确率

## 讨论

本研究展示了allo-HSCT患者出血事件对预后的判断价值。共有54.4％的造血干细胞移植患者在移植后发生出血事件。Ⅲ度以上出血与OS率以及NRM均显著相关。相比之下，出血与CIR无明显相关性。此外，Ⅲ度以上出血与不良后果独立相关，并在识别高风险患者方面具有显著优势。我们构建的出血风险模型包括基本的供者合并心脑血管疾病、GVHD分度、血小板重建、血小板输注无效和尿常规异常，成功预测了造血干细胞移植患者的出血风险。

出血虽然对恶性血液病的进展和复发没有明显影响，但能够提高患病群体的NRM，可以导致较差的临床结局。考虑到造血干细胞移植患者出血事件的高发生率[5]，有效控制出血可能是改善造血干细胞移植患者预后的关键。虽然预防性输注血小板和补充凝血因子可以改善出血风险，但常规疗法对一部分严重出血的病例无效，使其死亡风险增加[6]。因此，确定血小板减少和凝血功能以外的出血风险因素，并且对此进行干预和预防，是提高allo-HSCT患者长期生存率的有效手段。

我们用Lasso回归法筛选出与出血事件相关的因素，这是一种控制串联性的机器学习方法[7]。包括整个队列75％的训练集结果显示，供者合并心脑血管疾病、GVHD分度、血小板重建不良、血小板输注无效和尿常规异常是严重出血的独立风险因素。Labrador等[8]研究显示，晚期疾病、消融性调理方案、脐带血移植、造血干细胞移植后抗凝、Ⅲ/Ⅳ级GVHD以及TMA与出血事件有关，抗凝治疗、严重血小板减少与造血干细胞移植后晚期出血有关。Gerber等[9]的一项研究显示，抗凝、人种因素、肝静脉闭塞病和GVHD与出血风险增加呈正相关。我们没有评估抗凝治疗的影响，因为在我们的病例中，肝素是预处理过程中的常规用药。由于发病率低，肝静脉闭塞病和血栓性微血管病对出血事件没有显示出明显的预测价值。我们的队列和其他研究之间的出血风险因素的主要差异可能归因于以下原因。首先，不同研究中的移植方法以及预处理策略是不同的。第二，种族群体不同。第三，各研究的随访时间不同。但我们的研究不仅包括更大的样本量，而且还进行了交叉验证，以及使用引导抽样法进行内部验证。此外，我们使用多个机器学习模型对常规Logistics回归的结果进行比较，并使用决策曲线分析评估出血评分的预后价值，探讨了其临床意义。但本研究仍然属于回顾性研究，对于造血干细胞移植后出血因素的判定并且对出血进行有效预测，需要开展多中心大样本的前瞻性研究来验证。

严重的血小板减少被认为是造血干细胞移植后出血的一个重要因素[10]，也是凝血障碍的一个主要指标[11]。预防性血小板输注已被证明可以降低造血干细胞移植后长期严重血小板减少患者的出血的发生率[12]。然而，患者可能对外源性血小板输注产生抵抗，并出现难治性出血。另一方面，严重的GVHD可能会通过损害血小板生成和促进血小板清除而加重血小板减少和血小板无效输注[13]。此外，我们的研究显示供者合并心脑血管疾病是造血干细胞移植后出血的一个新的风险因素。严格选择供者可能通过减少出血风险来改善造血干细胞移植患者的预后。严重的GVHD可能导致造血和血管系统的弥漫性损伤，血栓性微血管病或肝静脉闭塞病发病后继发性低凝状态也可能是造血干细胞移植后出血的基础。我们的队列中这两类血栓性疾病的发生率要低得多。这一差异可能是由于在造血干细胞移植前强制服用肝素所致。未来有必要进行多中心和大规模的前瞻性研究，以确定抗凝药物在预防和治疗造血干细胞移植后出血中的价值。

本研究结果证实，GVHD是造成造血干细胞移植后出血的主要因素。同时，GVHD也是造血干细胞移植患者不良的预后因素。横断面研究显示，急性GVHD患者的出血风险更高[10],[13]–[14]，而且GVHD的分度与出血的严重程度相关[10],[14]。Ⅲ/Ⅳ级急性GVHD与颅内出血、胃肠道出血和肺出血有关[14]–[15]。

出血作为造血干细胞移植后的一个致命事件，需要早期预测和干预，以减少继发性不良事件。据我们所知，目前还没有完整的造血干细胞移植出血评分。MOBRI、HAS-BLED和HEMORR2等评分已被应用于心血管相关疾病患者出血的评估。鉴于接受造血干细胞移植的患者和心血管疾病患者之间的综合差异，这些评分对造血干细胞移植后出血的预测作用相对较差。我们建立了一个新的出血评分模型，纳入与造血干细胞移植相关的5个因素，与其他出血评分系统相比，我们的模型在预测出血风险方面有更好的估计临床效益。不同的机器学习模型显示了与传统逻辑模型一致的诊断效率。新评分不仅对出血有预测作用，而且还显示出对造血干细胞移植患者的预后价值。

本研究结果显示，造血干细胞移植后出血增加了总体OM和NRM。出血等级在造血干细胞移植患者中可提供超越其他因素的额外预测能力。交叉验证的多变量评分显示出血等级与预测造血干细胞移植患者长期预后的功效非常吻合。
